# Supplementary material for: Predicting visual function by interpreting a neuronal wiring diagram
Source: Nature. 2024 Oct 2;634(8032):113–23. doi: 10.1038/s41586-024-07953-5 (PMC11446822; doi:10.1038/s41586-024-07953-5)
Supplement: Supplementary file 6 — CRF and ERF predictions for individual TmY4 and TmY9 cells. Analogous to Supplementary Data 3, but for TmY target types. Shown are the top four monosynaptic pathways, the strongest pathway passing through each of the top ten intermediary types (ranking from Extended Data Fig. 7), and the trisynaptic pathway Tm1–TmY–Dm3–TmY (see the section entitled Prediction of spatial normalization). [file 41586_2024_7953_MOESM6_ESM.zip › DataS4/TmY9q/720575940648211076.pdf]

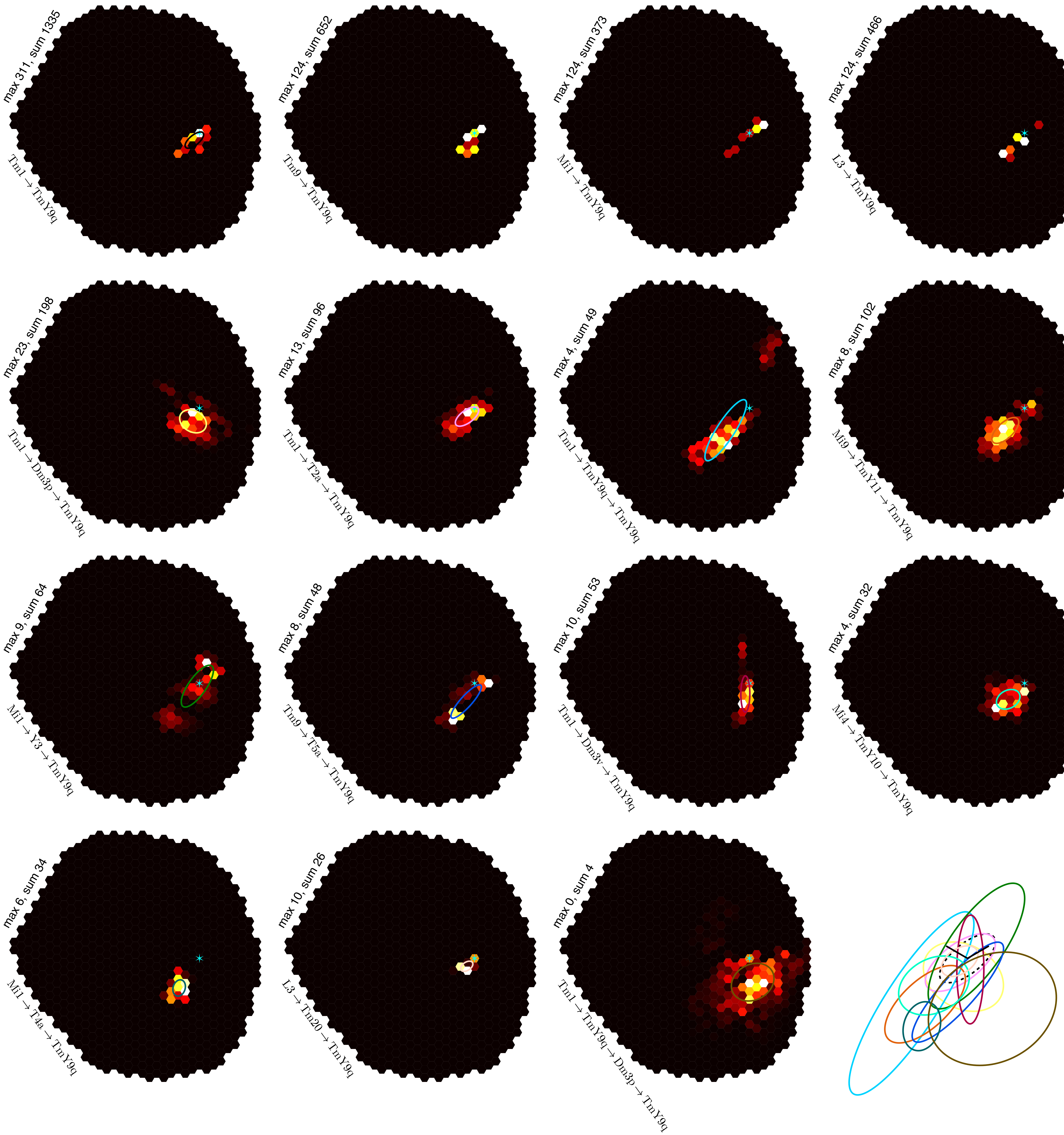
max 311, sum 1335
Tm1 → TmY9q
max 124, sum 652
Tm9 → TmY9q
max 124, sum 373
Mi1 → TmY9q
max 124, sum 466
L3 → TmY9q
max 23, sum 198
Tm1 → Dm3p → TmY9q
max 13, sum 96
Tm1 → T2a → TmY9q
max 4, sum 49
Tm1 → TmY9q → TmY9q
max 8, sum 102
Mi9 → TmY11 → TmY9q
max 9, sum 64
Mi1 → Y3 → TmY9q
max 8, sum 48
Tm9 → T5a → TmY9q
max 10, sum 53
Tm1 → Dm3v → TmY9q
max 4, sum 32
Mi4 → TmY10 → TmY9q
max 6, sum 34
Mi1 → T4a → TmY9q
max 10, sum 26
L3 → Tm20 → TmY9q
max 0, sum 4
Tm1 → TmY9q → Dm3p → TmY9q
